# Glucose versus fructose metabolism in the liver measured with deuterium metabolic imaging

**DOI:** 10.3389/fphys.2023.1198578

**Published:** 2023-07-03

**Authors:** Arjan D. Hendriks, Andor Veltien, Ingmar J. Voogt, Arend Heerschap, Tom W. J. Scheenen, Jeanine J. Prompers

**Affiliations:** ^1^ Center of Image Sciences, University Medical Center Utrecht, Utrecht, Netherlands; ^2^ Department of Medical Imaging (Radiology), Radboud University Medical Center, Nijmegen, Netherlands; ^3^ WaveTronica BV, Utrecht, Netherlands

**Keywords:** liver, metabolism, glucose, fructose, magnetic resonance spectroscopy, deuterium, hepatic steatosis

## Abstract

Chronic intake of high amounts of fructose has been linked to the development of metabolic disorders, which has been attributed to the almost complete clearance of fructose by the liver. However, direct measurement of hepatic fructose uptake is complicated by the fact that the portal vein is difficult to access. Here we present a new, non-invasive method to measure hepatic fructose uptake and metabolism with the use of deuterium metabolic imaging (DMI) upon administration of [6,6’-^2^H_2_]fructose. Using both [6,6’-^2^H_2_]glucose and [6,6’-^2^H_2_]fructose, we determined differences in the uptake and metabolism of glucose and fructose in the mouse liver with dynamic DMI. The deuterated compounds were administered either by fast intravenous (IV) bolus injection or by slow IV infusion. Directly after IV bolus injection of [6,6’-^2^H_2_]fructose, a more than two-fold higher initial uptake and subsequent 2.5-fold faster decay of fructose was observed in the mouse liver as compared to that of glucose after bolus injection of [6,6’-^2^H_2_]glucose. In contrast, after slow IV infusion of fructose, the ^2^H fructose/glucose signal maximum in liver spectra was lower compared to the ^2^H glucose signal maximum after slow infusion of glucose. With both bolus injection and slow infusion protocols, deuterium labeling of water was faster with fructose than with glucose. These observations are in line with a higher extraction and faster turnover of fructose in the liver, as compared with glucose. DMI with [6,6’-^2^H_2_]glucose and [6,6’-^2^H_2_]fructose could potentially contribute to a better understanding of healthy human liver metabolism and aberrations in metabolic diseases.

## Introduction

The increase in consumption of dietary sugars during the past decades has been associated with an increased prevalence of obesity and its metabolic complications (Bray et al., 2004; Malik et al., 2010; Johnson et al., 2017). In particular, chronic intake of high amounts of fructose, for example, by excessive consumption of high-fructose corn syrup-sweetened beverages, has been linked to the development of obesity, hepatic steatosis, hypertension, insulin resistance and diabetes, and Alzheimer’s disease (Stanhope et al., 2009; Tappy and Le, 2010; Maersk et al., 2012; Stanhope et al., 2015; Softic et al., 2020; Herman and Birnbaum, 2021; Malik and Hu, 2022; Johnson et al., 2023). However, for moderate fructose consumption, there is no solid evidence of its deleterious effects (Tappy and Le, 2012; Chiu et al., 2014; Chung et al., 2014).

The (supposedly) more harmful effects of fructose as compared to glucose may originate from differences in fructose versus glucose metabolism. In contrast to hepatic glucose uptake, the uptake of fructose is not regulated by hepatic energy status and the majority of fructose arriving at the liver via the portal vein is readily extracted by the liver (Tappy and Le, 2012; Hannou et al., 2018; Francey et al., 2019; Herman and Birnbaum, 2021). After rapid phosphorylation of fructose into fructose-1-phosphate, it is converted to triose phosphates, which are routed toward glucose and glycogen production, lactate production, or *de novo* lipogenesis, depending on the energy status of the liver (Tappy and Le, 2012; Hannou et al., 2018; Herman and Birnbaum, 2021). The lipogenic effect of fructose has been suggested to play a role in fructose-induced hepatic steatosis (Nunes et al., 2014; Softic et al., 2016; Janssens et al., 2017; Ter Horst and Serlie, 2017).

From an oral glucose load, 15%–35% is taken up by the liver and the remainder by peripheral tissues (Moore et al., 2012; Tappy and Le, 2012; Hannou et al., 2018). For the last half century, it was believed that an oral fructose load is almost completely (>70%) extracted by the liver (Tappy and Le, 2012; Hannou et al., 2018). However, this view has been challenged recently by experiments in mice, showing that at low doses of fructose, ∼90% of the fructose is cleared and converted to glucose and organic acids by the small intestine, resulting in very low concentrations of fructose, but high levels of fructose-derived glucose and organic acids (e.g., lactate) in the portal blood (Jang et al., 2018). Yet, high fructose loads exceeded the clearance capacity of the small intestine, resulting in spillover of fructose to the liver (Jang et al., 2018). Currently, the contribution of intestinal versus hepatic fructose clearance in humans is not clear (Herman and Birnbaum, 2021).

Direct measurement of hepatic glucose or fructose uptake is complicated by the fact that the portal vein is difficult to access (DeFronzo, 1987; Moore et al., 2012). Therefore, hepatic glucose/fructose uptake has typically been estimated from splanchnic balance measurements and tracer techniques (Radziuk et al., 1978; Katz et al., 1983; DeFronzo, 1987; Moore et al., 2012). However, considering the recent finding that the small intestine may play an important role in fructose metabolism, a more direct assessment of hepatic metabolism is warranted. Using ^13^C magnetic resonance spectroscopy (MRS), hepatic glucose uptake can be studied directly and non-invasively after intravenous or oral administration of [1–^13^C]glucose (Beckmann et al., 1993; Roden et al., 2001; Gursan and Prompers, 2022). Because of the large chemical shift range, signals in ^13^C MR spectra are usually very well resolved. However, ^13^C MRS has a low intrinsic sensitivity and ^13^C-enriched substrates are very costly (Gursan and Prompers, 2022). Recently, deuterium metabolic imaging (DMI) has emerged as a new technique to measure metabolism *in vivo* (De Feyter et al., 2018). Deuterium (^2^H) nuclei have much shorter T_1_ and T_2_ relaxation times compared to ^13^C nuclei (De Feyter et al., 2018; De Feyter and de Graaf, 2021). The shorter T_2_ relaxation times result in broader resonance lines and thus a lower spectral resolution of ^2^H compared with ^13^C MR spectra. However, in return, the shorter T_1_ relaxation times of ^2^H nuclei allow faster signal averaging, yielding a higher sensitivity per unit of time for DMI compared with ^13^C MRS. In addition, deuterium-labeled compounds are generally cheaper than their ^13^C-labeled counterparts. Using deuterium-labeled glucose ([6,6’-^2^H_2_]glucose), DMI has been used to measure glucose uptake and metabolism in the liver (De Feyter et al., 2018; De Feyter et al., 2021; Gursan et al., 2023).

In this study, we demonstrate the application of deuterium-labeled fructose ([6,6’-^2^H_2_]fructose) as a new substrate for DMI. Using both [6,6’-^2^H_2_]glucose and [6,6’-^2^H_2_]fructose, we aimed to measure differences in the uptake and metabolism of glucose and fructose in the mouse liver with dynamic DMI. This new application of DMI should provide more insight into the role of the liver in glucose versus fructose clearance.

## Materials and methods

Female C57BL/6J mice (20 ± 2 g, 9 weeks of age) were purchased from Charles River (Sulzfeld, Germany). The mice were housed at 21°C ± 1°C, 40%–50% humidity, and a 12:12 h light:dark cycle, with *ad libitum* access to water and standard rodent chow diet. The animal experiments were approved by the Central Animal Experiments Committee (CCD) of the Netherlands and the local animal welfare body (RU-DEC-2016-0013-011).

Magnetic resonance (MR) measurements were performed on an 11.7 T BioSpec Avance III small animal MR system, operating on the ParaVision 6.0.1 software platform (Bruker BioSpin, Ettlingen, Germany). Anatomical MR imaging (MRI) and DMI were performed with a standard Bruker ^1^H transmit-receive body coil (diameter: 72 mm; frequency: 500 MHz) combined with a custom-built ^2^H transmit-receive elliptical surface coil (dimensions: 16 mm × 20 mm; frequency: 76.8 MHz). Before the experiments, the animals were sedated in a chamber with 4% isoflurane in a 2:1 medical air and O_2_ mixture. During the measurements, anesthesia was maintained at 1%–2% isoflurane through a customized face mask. Body temperature was monitored with a rectal temperature probe and maintained at 37°C with heated air flow. Respiration of the animal was monitored using a pneumatic cushion respiratory monitoring system (Small Animal Instruments Inc., NY, United States) and was maintained at 60-80 bpm by regulating the isoflurane levels throughout the experiment.

In total, 8 mice were scanned. Four mice received [6,6’-^2^H_2_]glucose (Merck Life Science NV, Amsterdam, Netherlands), and four mice received [6,6’-^2^H_2_]fructose (CortecNet, Paris-Saclay, France), at a dose of 1.3 g of deuterated substrate per kg body weight, dissolved in a saline solution so that the injected volume was 0.1 mL solution per 30 g body weight (at a concentration of 2.1 M for both [6,6’-^2^H_2_]glucose and [6,6’-^2^H_2_]fructose). The glucose/fructose load was administered via either a fast (∼1 s) intravenous (IV) tail vein bolus injection (1 mouse with glucose, and 1 mouse with fructose), or a slow IV tail vein infusion at 2.3 μL/min for ∼30 min (3 mice with glucose, and 3 mice with fructose).

The MR scan protocol consisted of ^1^H anatomical MRI (T_1_ weighted 3D FLASH, field of view (FOV) = 33 × 33 × 33 mm^3^, matrix = 108 × 108 × 108), B_0_ shimming of a voxel of approximately 3 × 5 × 5 mm^3^ in the liver, and a natural abundance 3D DMI scan for reference. At the start of infusion, a time series of dynamic 3D DMI scans was started with a total duration of either 60 min (bolus injection) or 90 min (slow infusion). DMI measurements were performed with a 3D MR spectroscopic imaging sequence (Brown et al., 1982) with spherical *k*-space encoding. DMI scan parameters were as follows: FOV = 33 × 33 × 33 mm^3^, matrix = 9 × 9 × 9, voxel size = 3.7 × 3.7 × 3.7 mm^3^, repetition time (TR) = 250 ms, flip angle = 90°, acquisition delay = 0.63 ms, spectral bandwidth = 5,000 Hz, 1,024 data points, 2 averages, and scan duration = 3:00 min. In addition to the animal experiments, DMI experiments were performed on vials with the [6,6’-^2^H_2_]glucose and [6,6’-^2^H_2_]fructose solutions, using the same protocol.

Processing of the 3D DMI datasets was done with DMIWizard v1.1 (De Feyter et al., 2018), and included 2 Hz Lorentzian apodization and zero filling to 2048 points. For each animal experiment, a single voxel, centrally located in the liver, was selected from the 3D DMI datasets for further analyses. Signals of deuterated water (HDO; 4.79 ppm) and [6,6’-^2^H_2_]glucose/[6,6’-^2^H_2_]fructose (fitted by a single resonance line at 3.8 ppm) in the deuterium spectra were fitted in the time domain using a nonlinear least squares algorithm (advanced method for accurate, robust, and efficient spectral fitting; AMARES) in the jMRUI software package (Vanhamme et al., 1997; Stefan et al., 2009), assuming Lorentzian line shapes. Tissue glucose/fructose concentrations were calculated with reference to the baseline naturally abundant HDO signal integral, which was set to represent 13.7 mM HDO, and taking into account the number of deuterons (Veltien et al., 2021).

Tissue glucose/fructose concentrations after bolus injection of glucose and fructose (excluding the first data point) were fitted with a mono-exponential function using GraphPad Prism version 9.5.0 for Windows (GraphPad Software, San Diego, CA, United States). For the data obtained with slow infusion of glucose or fructose, significant differences between the glucose/fructose and deuterated water signal amplitudes were assessed with a two-way ANOVA for repeated measures with one within-subjects factor (time) and one between-subjects factor (group: glucose versus fructose infusion), with Bonferroni *post hoc* analyses. Statistical analyses were performed in IBM SPSS Statistics 27.0 (SPSS Inc., Chicago, IL, United States) and significance was set at *p* < 0.05.

## Results

**FIGURE 1:**
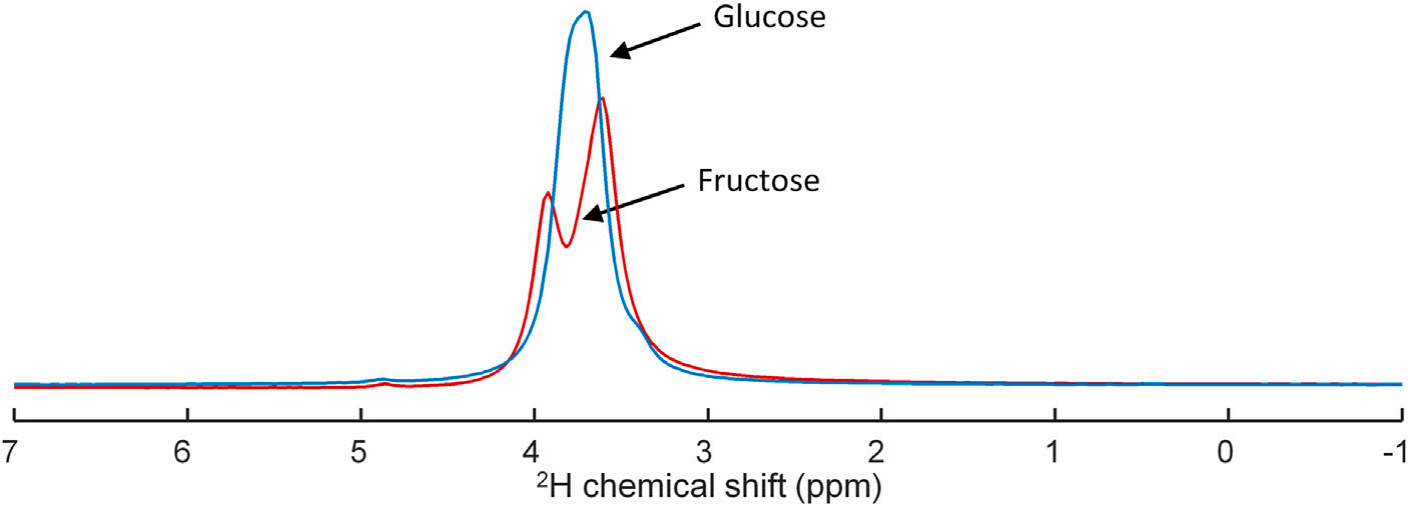
Superposition of deuterium (^2^H) MR spectra obtained of tubes containing either [6,6’-^2^H_2_]glucose (blue) or [6,6’-^2^H_2_]fructose (red) in concentrations of 2.1 M, demonstrating overlap of the resonances of glucose and fructose and their different peak shape.

Separate deuterium MR spectra taken from the [6,6’-^2^H_2_]glucose and [6,6’-^2^H_2_]fructose solutions, which were used in the animal experiments, showed different spectroscopic patterns for these compounds (Figure 1). In contrast to a single, slightly broadened resonance of deuterated glucose centered at ∼3.8 ppm, deuterated fructose yielded a signal centered at ∼3.7 ppm and a partially resolved smaller signal centered at ∼4.0 ppm.

**FIGURE 2:**
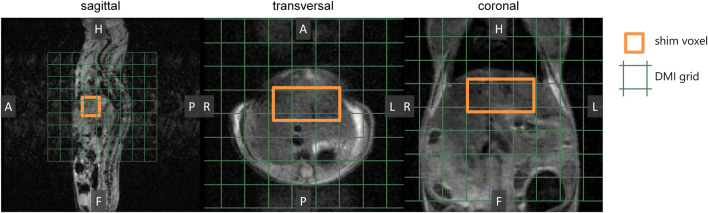
Preparation of DMI measurements. *In vivo* anatomical 3D MR images were acquired as a guide to perform B_0_ shimming on a voxel (3 × 5 × 5 mm^3^; orange) and to position the DMI grid (green).

**FIGURE 3:**
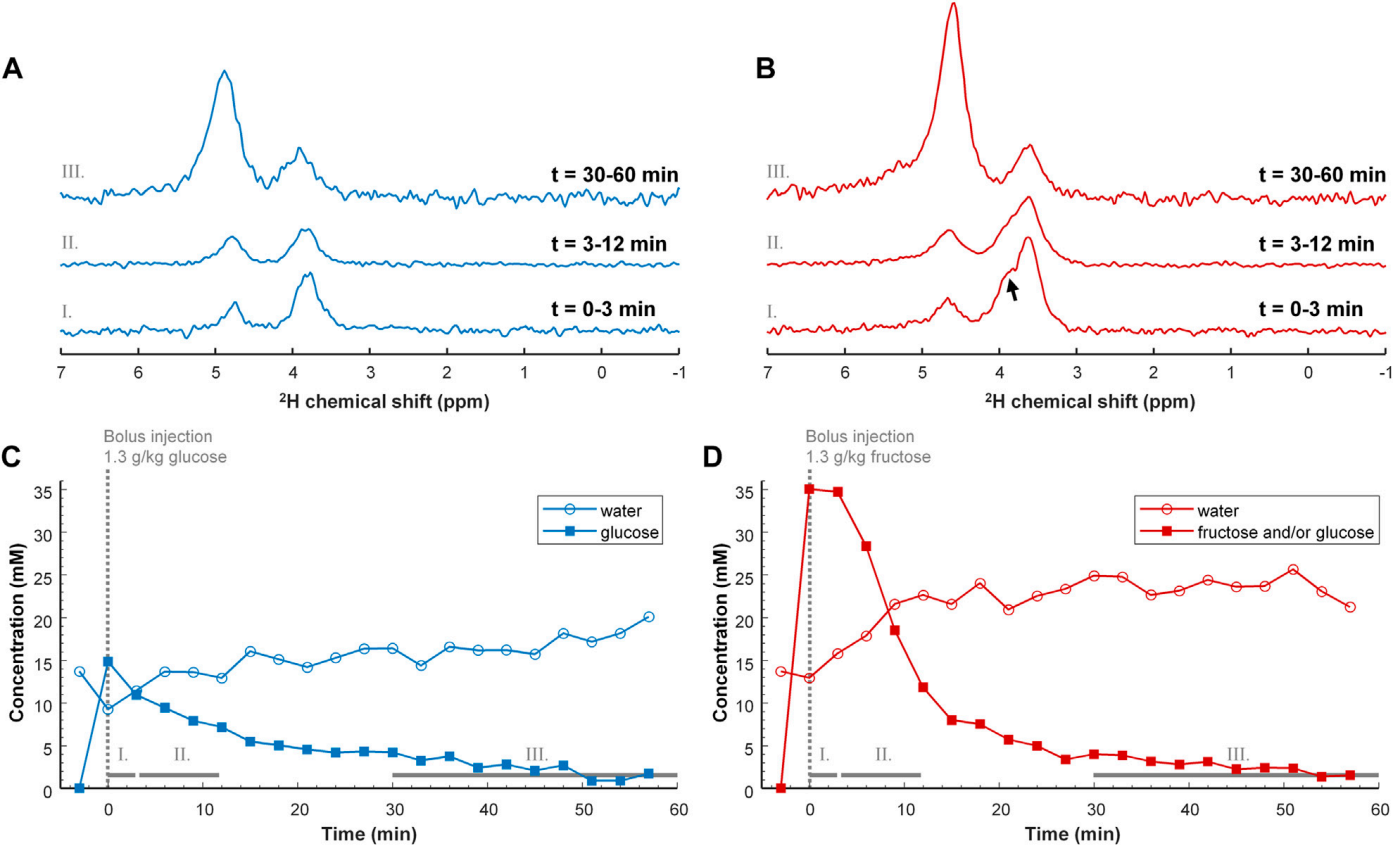
Top **(A,B)** DMI spectra of the liver after IV bolus injection of 1.3 g/kg [6,6’-^2^H_2_]glucose (A; blue) and [6,6’-^2^H_2_]fructose (B; red). The DMI spectra were obtained after averaging over a period of: 0–3 min (I, bottom), 3–12 min (II, middle) and 30–60 min (III, top). Directly after injection of deuterated fructose, the spectroscopic peak of fructose is broader than that of glucose and shows a shoulder (arrow), which is also present in the separate measurement of the [6,6’-^2^H_2_]fructose solution (Figure 1). The shoulder has disappeared after 30 min, supporting the concept of conversion of fructose to glucose. The spectra in trace III are amplified by 5 with respect to those in I and II. Bottom **(C,D)** Tissue concentration curves of deuterated glucose and/or fructose, and of deuterated water as a function of time after IV bolus injection of 1.3 g/kg [6,6’-^2^H_2_]glucose (C; blue) and [6,6’-^2^H_2_]fructose [**(D)**; red]. Note that the tissue concentration of deuterated glucose and/or fructose, represented by the 3.8 ppm signal [square symbols in panels **(C,D)**], is much higher immediately after injection of fructose compared to glucose. Despite this large initial difference, the tissue concentration seems to return to the same value at around 30 min after bolus injection of either glucose or fructose.

For the mouse experiments, we first obtained anatomical MR images to guide the location of a voxel in the liver for B_0_ shimming, and planning of the DMI grid (Figure 2). After injection of a bolus of [6,6’-^2^H_2_]glucose or [6,6’-^2^H_2_]fructose, repeated *in vivo* 3D DMI measurements covering the liver resulted in a time series of ^2^H MR spectra with signals from water and glucose or fructose (Figures 3A,B). Although the resonance lines of the *in vivo* deuterium MR spectra were broader than those of the [6,6’-^2^H_2_]glucose and [6,6’-^2^H_2_]fructose solutions, during the first few minutes (0–3 min) after bolus injection of [6,6’-^2^H_2_]fructose, the spectroscopic pattern of [6,6’-^2^H_2_]fructose (larger signal at ∼3.7 ppm and smaller signal at ∼4.0 ppm) could be recognized in the ^2^H MR spectra of the liver (Figure 3B). During later time points (30–60 min), only a single peak appeared to be present at ∼3.8 ppm, similar to the spectra after bolus injection of [6,6’-^2^H_2_]glucose (Figures 3A,B). Because the two fructose signals were not resolved and because the spectroscopic pattern changed as a function of time after bolus injection of fructose, for quantification of the spectroscopic data of the dynamic experiments (Figures 3C,D) only a single resonance line was fit for either fructose or glucose at 3.8 ppm. This quantitative analysis revealed that the initial tissue concentration of [6,6’-^2^H_2_]fructose after bolus injection was more than two-fold higher than that of [6,6’-^2^H_2_]glucose after bolus injection (35 versus 15 mM), while the amount of injected substrate was the same for both experiments (i.e., 1.3 g per kg body weight). The decay of the 3.8 ppm signal during the following time points was fitted with a mono-exponential function, yielding time constants of 19.8 and 8.0 s for the experiments with glucose and fructose, respectively, i.e., a 2.5-fold faster decay after fructose injection. The initial rise of the deuterated water signal was also faster after fructose injection than after glucose injection. After about 20 min, the concentration curve of the 3.8 ppm signal was very similar for the experiments with glucose and fructose injection. From that time point on, the increase in deuterated water was also similarly slow for the experiments with both glucose and fructose.

**FIGURE 4:**
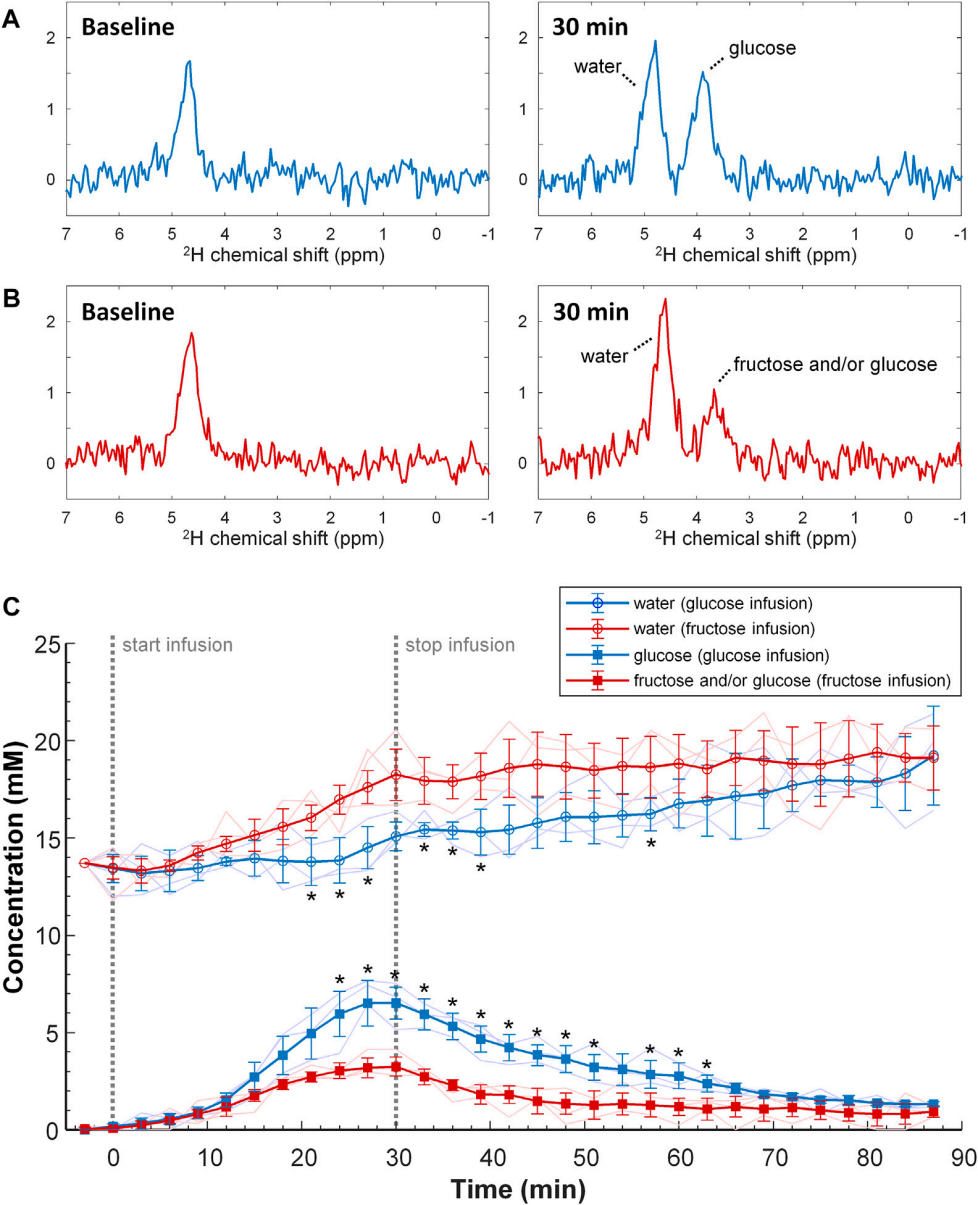
Top and middle **(A,B)** Individual DMI spectra obtained of the liver at baseline (left) and at the end (t = 30 min; right) of slow IV infusion of 1.3 g/kg [6,6’-^2^H_2_]glucose [**(A)**; blue] and [6,6’-^2^H_2_]fructose [**(B)**; red] over 30 min. Bottom **(C)** Tissue concentration of deuterated glucose and/or fructose, and of deuterated water as a function of time during and after slow IV infusion of 1.3 g/kg [6,6’-^2^H_2_]glucose (blue) and [6,6’-^2^H_2_]fructose (red). The mean and standard deviation of the concentrations over 3 mice with a moving average filter is displayed, including their individual time curves (thin lines). The start and stop of the 30 min infusion period is indicated (dashed grey lines). Note that, during infusion of fructose compared to glucose, the 3.8 ppm peak has a slower increase whereas the tissue concentration of deuterated water is increasing faster. *p < 0.05 versus the group with fructose infusion, for the same signal and time point.

In the DMI experiments with a 30 min slow infusion of [6,6’-^2^H_2_]glucose or with the same amount of [6,6’-^2^H_2_]fructose, again a signal appeared at ∼3.8 ppm in the ^2^H MR spectra of the liver. Due to the much lower signal amplitudes compared to the experiments with bolus injection, it was not possible to differentiate between the spectroscopic patterns of [6,6’-^2^H_2_]fructose and [6,6’-^2^H_2_]glucose upon slow infusion of [6,6’-^2^H_2_]fructose. In contrast to the bolus injection experiments, the signal at ∼3.8 ppm was clearly higher for glucose than for fructose administration (Figures 4A,B). An analysis of the spectra as a function of time, averaged over the different animals (n = 3 per group), revealed a gradual increase in the 3.8 ppm signal during the 30 min infusion period, followed by a slow decrease after the infusion had stopped (Figure 4C). However, with slow infusion of [6,6’-^2^H_2_]fructose, the amplitude of the 3.8 ppm signal was significantly lower compared to that with [6,6’-^2^H_2_]glucose infusion for the time points 24–63 min after the start of infusion (except for the 54 min time point). The deuterated water signal slowly increased during [6,6’-^2^H_2_]glucose infusion and kept increasing after the infusion had stopped. In comparison, during [6,6’-^2^H_2_]fructose infusion the deuterated water concentration increased more rapidly, but it slowed down after the end of the infusion (Figure 4C). At the final time point, 60 min after the end of the infusions of [6,6’-^2^H_2_]glucose and [6,6’-^2^H_2_]fructose, the tissue concentrations of deuterated glucose and/or fructose, represented by the signal at 3.8 ppm, and of deuterated water were very similar for [6,6’-^2^H_2_]glucose and [6,6’-^2^H_2_]fructose infusion.

## Discussion

Here we present a new application of DMI with administration of [6,6’-^2^H_2_]fructose to measure hepatic fructose uptake and metabolism. Directly after IV bolus injection of [6,6’-^2^H_2_]fructose, a much higher fructose uptake was observed in the mouse liver as compared to that of glucose after bolus injection of [6,6’-^2^H_2_]glucose. This is in line with literature describing that fructose is almost completely extracted by the liver, whereas glucose is also consumed by other tissues (Tappy and Le, 2012; Hannou et al., 2018). The higher uptake of fructose was associated with a much faster decay of its ^2^H signal than that of glucose in spectra of the liver during the first ∼10 min after bolus injection. In contrast, after slow IV infusion of fructose, the ^2^H fructose and/or glucose signal maximum in liver spectra was lower compared with ^2^H glucose signal maximum after slow infusion of glucose. These observations can be reconciled with the high turnover of fructose in the liver (Tappy and Le, 2012; Hannou et al., 2018; Francey et al., 2019; Herman and Birnbaum, 2021).

Deuterium MR spectra from [6,6’-^2^H_2_]glucose and [6,6’-^2^H_2_]fructose solutions showed that their ^2^H signals overlap, but have a clearly different shape (Figure 1). Whereas for [6,6’-^2^H_2_]glucose a single, unresolved resonance, centered at ∼3.8 ppm, was observed, for fructose a main signal centered at ∼3.7 ppm and a smaller signal centered at ∼4.0 ppm could be distinguished. In solution, fructose is an equilibrium mixture of 5 tautomers, of which the dominating ones are β-fructopyranose (∼70%), β-fructofuranose (∼23%), and α-fructofuranose (∼6%). The ^1^H-6 and ^1^H-6’ spins of β-fructopyranose resonate ∼0.3 ppm apart at about 4.0 and 3.7 ppm and those of the other tautomers resonate ∼0.1 ppm apart at about 3.8 and 3.7 ppm (Barclay et al., 2012). Therefore, we assign the peak at ∼4.0 ppm in the ^2^H MR spectrum of the [6,6’-^2^H_2_]fructose solution to the ^2^H-6 of β-fructopyranose and the larger peak at ∼3.7 ppm to the ^2^H-6’ of β-fructopyranose and the deuterons of the other fructose anomers. In the *in vivo* liver DMI spectra, a resonance emerged at ∼3.8 ppm right after IV bolus injection of both [6,6’-^2^H_2_]glucose and [6,6’-^2^H_2_]fructose. However, from the peak shapes, the spectroscopic pattern of [6,6’-^2^H_2_]fructose, with a distinct shoulder at ∼4.0 ppm, could be recognized in the spectra during the first minutes after bolus injection of [6,6’-^2^H_2_]fructose (Figure 3B). During later time points (30–60 min), this shoulder seemed to disappear and only a single peak was observed at ∼3.8 ppm, similar to the spectra after bolus injection of [6,6’-^2^H_2_]glucose, supporting the rapid conversion of fructose into glucose (Tappy and Le, 2012; Hannou et al., 2018; Herman and Birnbaum, 2021).

During the first ∼10 min after IV bolus injection of [6,6’-^2^H_2_]fructose, the 3.8 ppm signal in the *in vivo* liver DMI spectra decayed much faster than after bolus injection of [6,6’-^2^H_2_]glucose. In addition, the deuterated water concentration increased more rapidly during the first ∼10 min after bolus injection of [6,6’-^2^H_2_]fructose. In contrast to the experiments with bolus injections, after slow IV infusion of [6,6’-^2^H_2_]fructose, the maximum of the 3.8 ppm signal was lower compared with that after slow infusion of [6,6’-^2^H_2_]glucose. However, also during slow infusion, the deuterated water concentration increased more rapidly with [6,6’-^2^H_2_]fructose as compared with [6,6’-^2^H_2_]glucose. After uptake in the liver, fructose is instantly phosphorylated into fructose-1-phosphate, which is then metabolized into triose phosphates. In contrast to glucose, the hepatic uptake of fructose and its metabolism into triose phosphates is not regulated by the hepatic energy status (Tappy and Le, 2012; Hannou et al., 2018; Francey et al., 2019; Herman and Birnbaum, 2021). The consequent higher turnover of fructose in the liver as compared to glucose could explain the faster decay of its ^2^H signal in the bolus measurement, the lower signal maximum in the slow infusion measurements, and the faster increases in tissue concentration of deuterated water with both protocols. However, the deuterated water is not necessarily produced in the liver itself, but likely originates (also) from other tissues, such as the skeletal muscles, heart and brain, especially for the later time points.

Fructose-derived triose phosphates in the liver are routed toward glucose and glycogen production, lactate production, or *de novo* lipogenesis, depending on the hepatic energy status (Tappy and Le, 2012; Hannou et al., 2018; Herman and Birnbaum, 2021). The peak shapes in our *in vivo* liver DMI spectra support the conversion of fructose to glucose in the liver after IV bolus injection of [6,6’-^2^H_2_]fructose. However, signals from deuterated lactate or lipids (with overlapping resonance frequencies) were not detected. The decrease of the 3.8 ppm signal arising from the liver after IV bolus injection and after slow infusion of both [6,6’-^2^H_2_]fructose and [6,6’-^2^H_2_]glucose is most likely explained by its conversion into (^2^H MRS invisible (De Feyter et al., 2021)) glycogen and by hepatic glucose release (Moore et al., 2012; Tappy and Le, 2012; Hannou et al., 2018; Herman and Birnbaum, 2021).

A limitation of our method is that part of the signal that we observe in the liver could also originate from blood. Liver blood volume is as much as 25–30 mL/100 g liver weight (Eipel et al., 2010). However, at the first time point after IV bolus injection, the plasma fructose concentration after bolus injection of [6,6’-^2^H_2_]fructose should be equal to the plasma glucose concentration after bolus injection of [6,6’-^2^H_2_]glucose, because we used equal amounts of [6,6’-^2^H_2_]fructose and [6,6’-^2^H_2_]glucose. Therefore, the higher 3.8 ppm signal directly after fructose bolus injection compared to glucose bolus injection cannot be attributed to differences in plasma fructose/glucose concentrations and should originate from a higher amount of fructose taken up by hepatocytes compared to the amount of glucose taken up by hepatocytes.

While it was commonly believed that the liver is the main site of fructose metabolism (Tappy and Le, 2012; Hannou et al., 2018), Jang et al. recently showed that in mice the small intestine clears most dietary fructose when consumed at low doses, whereas high fructose doses spill over to the liver (Jang et al., 2018). In our study, fructose and glucose were administered intravenously, but it would be interesting to acquire *in vivo* DMI data after oral [6,6’-^2^H_2_]fructose and [6,6’-^2^H_2_]glucose administration and compare the results. For the application of DMI in humans, deuterated substrates are usually administered via the oral route and by using [6,6’-^2^H_2_]fructose, this could provide insight into intestinal versus hepatic fructose clearance.

Limitations of this study include the low number of animals and the lack of *ex vivo* measurements of liver gene and/or protein expression and metabolite concentrations. For the experiments with IV bolus injection, we used one animal per condition (i.e., glucose versus fructose injection), which precludes the assessment of inter-individual variability. However, the inter-individual variabilities after slow IV infusion of either glucose or fructose (Figure 4) were small compared to the group differences, signifying that the more than two-fold higher initial uptake of fructose versus glucose upon IV bolus injection will likely also be representative for larger group sizes. While *ex vivo* measurements of liver gene and/or protein expression and metabolite concentrations would be more relevant for studies on longer-term consumption of fructose versus glucose, it would have been interesting to determine the deuterium enrichment of the liver glycogen pool after injection/infusion, in order to assess differences in metabolic fate of glucose versus fructose within the liver. Deuterated glycogen is ^2^H MRS invisible *in vivo*, because of its large size and consequent short T_2_ relaxation time, but in isolated liver glycogen in solution the deuterium enrichment can be determined by ^2^H NMR upon addition of the glucose-releasing enzyme amyloglucosidase (De Feyter et al., 2021). Another important aspect of this study is that the DMI measurements were performed under isoflurane anesthesia. Isoflurane increases blood glucose concentrations in mice, without significantly affecting insulin secretion (Windelov et al., 2016), indicating reduced glucose uptake by peripheral tissues, which will probably be compensated by a higher hepatic glucose uptake. In contrast, hepatic fructose metabolism will likely be less affected by anesthesia. Therefore, the observed two-fold higher initial uptake of fructose versus glucose upon IV bolus injection in mice under isoflurane anesthesia may be an underestimation of what is expected in awake mice and humans. Similarly, the results obtained with slow IV infusion may have been affected by the isoflurane anesthesia, in particular for glucose infusion.

In conclusion, DMI with administration of [6,6’-^2^H_2_]fructose and [6,6’-^2^H_2_]glucose showed that in mice, hepatic uptake of fructose is more than two-fold higher than that of glucose after IV bolus injection. Moreover, differences in the kinetics of deuterium labeling after bolus injection of [6,6’-^2^H_2_]fructose and [6,6’-^2^H_2_]glucose and during slow infusion of [6,6’-^2^H_2_]fructose and [6,6’-^2^H_2_]glucose indicate a faster turnover of fructose in the liver. In the future, this method could potentially contribute to a better understanding of human liver metabolism, both in the healthy state and in diseases like diabetes and non-alcoholic fatty liver disease.

## Data Availability

The raw data supporting the conclusion of this article will be made available by the authors, without undue reservation.
